# Evaluation of Physicochemical Properties, Bioactive Composition, and Antioxidant Activity of *Prunus armeniaca* L. Cultivars for Functional Food and Nutraceutical Development

**DOI:** 10.3390/molecules31060988

**Published:** 2026-03-16

**Authors:** Ceren Birinci, Anna Kurek-Górecka, Elsevar Asadov, Zenon P. Czuba, Sevgi Kolaylı

**Affiliations:** 1 Department of Chemistry, Faculty of Science, Karadeniz Technical University, 61080 Trabzon, Türkiye; cerendidar.birinci@gmail.com; 2 Department of Microbiology and Immunology, Faculty of Medical Sciences in Zabrze, Medical University of Silesia in Katowice, Jordana 19, 41-808 Zabrze, Poland; zczuba@sum.edu.pl; 3 Nakhchivan State University, Nakhchivan AZ7012, Azerbaijan; asadoves1974@gmail.com

**Keywords:** apricot, antioxidant, phenolic, sugar, Malatya, Nakhchivan

## Abstract

Advancing the characterization of bioactive compounds in *Prunus armeniaca* L. is critical for identifying high-value cultivars with enhanced nutritional and functional potential. In this study, selected apricot varieties were evaluated, with particular emphasis on their sugar composition, phenolic profile, and antioxidant capacity. Sugar and phenolic compounds were analyzed using RP-HPLC-RID and RP-HPLC-PDA methods, respectively. Total phenolic content (TPC), total flavonoid content (TFC), and ascorbic acid levels were determined, and antioxidant activities were assessed using FRAP and DPPH assays. Distinct varietal differences were observed among the apricot cultivars. Sucrose, glucose, and fructose were identified as the dominant sugars contributing to the nutritional quality of the fruits. The phenolic composition was characterized by high levels of flavonoids and phenolic acids, which were strongly associated with antioxidant activity. Among the studied cultivars, Amasya (A-7) and Nakhchivan Adi Şalax (A-2) exhibited markedly higher total phenolic and flavonoid contents, as well as superior antioxidant capacities compared with other varieties. These findings demonstrate substantial biochemical variability among apricot cultivars and highlight A-7 and A-2 as promising candidates for functional food and nutraceutical applications due to their rich bioactive compound content and strong antioxidant potential.

## 1. Introduction

Apricot (*Prunus armeniaca* L.), a member of the Rosaceae family, is a widely cultivated fruit of considerable nutritional and economic importance. Although native to Central Asia, apricot cultivation has expanded globally, demonstrating adaptability to diverse climates, particularly temperate regions [1,2]. Apricot trees are adapted to growing in regions characterized by hot summers, cool winters, and high sunshine hours; however, climate conditions play a decisive role in yield and fruit quality, particularly due to their high sensitivity to late spring frosts. Differences in temperature, light intensity, and growing environment can significantly affect the accumulation of sugars, phenolic compounds, and other nutritional components in apricot fruits. Apricots are a widely cultivated fruit in Europe, the Middle East, and the Mediterranean basin. Today, Türkiye, Spain, Italy, Iran, China, and Azerbaijan are among the leading producing countries [3,4]. Among these countries, Türkiye holds a leading position globally, particularly in the production and export of dried apricots, thanks to its favorable agroclimatic conditions. The Malatya region, considered the center of apricot cultivation in Türkiye, plays a decisive role in the international market with its high-quality dried apricot production. The fruit is consumed fresh, dried, or processed into jams, juices, and oils, and is highly valued for its beneficial effects on intestinal health due to its rich fiber content and prebiotic sugars [5]. Owing to these nutritional attributes, along with its bioactive compound profile, apricot is also considered an important fruit with significant potential as a functional food. The diversity of apricot cultivars is notable, as they vary according to region and intended use. Prominent varieties include Hacıhaliloğlu, Şekerpare, Patterson, Moorpark, and Tilton. These cultivars exhibit varying attributes in terms of taste, texture, size, and durability, catering to diverse consumer preferences and industrial needs [1,3]. Malatya apricot is a globally renowned product with geographical indication; Malatya is often referred to as the “apricot capital of the world.” The region predominantly cultivates varieties such as Soğancı, Kabaaşı, Hasanbey, Hacıhaliloğlu, Çataloğlu, and Hacıkız. These cultivars are recognized for their exceptional quality and distinct flavor profiles, contributing significantly to Malatya’s esteemed position in the global apricot industry [5,6]. The Nakhchivan region is one of Azerbaijan’s main apricot growing centers due to its favorable climate and long-standing fruit-growing tradition. The region, dominated by a continental climate, experiences hot, dry summers and cold winters, significantly affecting apricot development and fruit quality. Local apricot genotypes are reported to be rich in sugars and phenolic compounds thanks to high sunshine duration and soil characteristics. However, scientific data on the physicochemical and biochemical properties of apricot varieties grown in Nakhchivan are still limited [7].

In terms of nutritional value, apricots are a low-calorie, high-fiber fruit that is highly beneficial for those following calorie-controlled diets. A 100 g serving of apricots provides approximately 45–50 kcal of energy and contains about 2 g of dietary fiber. This fiber content is reported to contribute to regulating energy intake and reducing the risk of overeating by increasing feelings of fullness [8,9]. Additionally, apricots are rich in essential nutrients such as potassium and vitamins A and C, which support overall health and metabolic function. Dried apricots maintain a similar nutritional profile, offering high levels of fiber, potassium, and phenolic compounds. Fresh and dried apricots are rich sources of vitamins A and C, potassium, iron, and dietary fiber. These nutrients contribute significantly to cardiovascular health and support the optimal functioning of the digestive and circulatory system [3,9].

Apricot is an economically and nutritionally important fruit, and, in Türkiye, Malatya and, in Azerbaijan, Nakhchivan are among the main apricot growing centers with long-standing production traditions. While the biochemical characteristics of Malatya apricot varieties have been studied in detail, physicochemical and nutritional data on apricots grown in Nakhchivan are limited. Furthermore, there are very few studies comparing these two neighboring production regions. This study aims to address this knowledge gap by comparatively evaluating the physical properties, sugar and phenolic compositions, and antioxidant capacities of apricot varieties from both regions [3,5,8].

## 2. Results and Discussion

### 2.1. Physicochemical Properties of Apricot Extracts

This study investigated the physical, chemical, and nutritional attributes of several apricot cultivars collected from geographically proximal regions located within two neighboring countries. The physicochemical properties of the fruits, including their mass, dimensional characteristics, and color parameters, are summarized in Table 1. The results indicated that the mean fruit weight varied between 42.03 and 74.10 g, while the average fruit length ranged from 41.33 to 57.00 mm. Furthermore, the mean seed weight of the examined cultivars was determined to be within the range of 2.23 to 3.39 g.

Minor variations in length, weight, and width were observed among the apricot samples. Although apricot fruit size is influenced by species, deriving definitive morphological characteristics from these measurements alone is not feasible, as they are also impacted by factors such as tree age, cultivation practices, and pruning and thinning techniques [1].

Nevertheless, it can be concluded that the A-1 and A-7 apricot cultivars are characterized by larger fruit sizes. Indeed, a study on Malatya apricots reported that the Hasanbey variety exhibited the highest average fruit weight compared to other cultivars. In the same study, the average weight of apricots from the Iğdır region was recorded as 32.33 ± 1.47 g. When compared with the values measured in the present study, significant differences were observed among the apricot cultivars [3]. A study conducted on 27 apricot genotypes from the Budapest region reported that fruit weights ranged from 22 to 78 g [8]. Similarly, variability was observed in kernel weight, with the A-4 apricot cultivar exhibiting the highest kernel weight, while the A-6 cultivar had the lowest. Apricot kernels, which are the seeds inside the hard pits of apricots, are highly valued for their nutritional and functional properties. Rich in oils, proteins, and bioactive compounds, they are used in various industries, including food, cosmetics, and traditional medicine. However, their consumption should be monitored due to the presence of amygdalin, which can release toxic cyanide when consumed in excessive amounts [10,11,12,13]. Dried apricot kernels, traditionally consumed as a snack after being sun-dried, are a popular food product in many cultures.

The soluble solid (SS) content of the apricot samples was found to range from 13.47% to 20.86%. The SS in apricots refers to the total amount of dissolved substances in the fruit, including sugars, organic acids, vitamins, and other soluble compounds. Soluble solid is also a crucial parameter for determining the sweetness and ripeness of apricots and is commonly used as an indicator of the fruit’s quality, influencing consumer preferences. Among the studied varieties, the highest SS value was found in the Hacıoğlu variety from the Malatya region (A-6), while the lowest value was observed in the Iğdır region apricot (A-5). No significant differences were observed among the apricot varieties from the Nakhchivan region apricots (from A-1 to A-4).

The external skin color of the fruits was quantified using CIE Lab color coordinates, revealing a color spectrum ranging from yellow to orange. The L* value of color ranged from 61 to 75, with no significant differences observed among the varieties, except for the A-7 variety. Although all apricot cultivars exhibit a light skin color, a comparison indicates that the A-7 cultivar differs significantly from the others. The CIE a* value (–a/+a) reflects the greenness and redness of the fruit, with our findings ranging from 13 to 26. Upon evaluation, it can be concluded that the A-4 cultivar shows the strongest greenish tone, while the A-7 cultivar reveals the most pronounced reddish hue. The b value (–b/+b) indicates the blueness and yellowness, with measurements ranging from 44 to 52. Very slight differences were observed between these color values. In a study conducted on 10 different apricot varieties, the L value ranged from 49 to 61, the a value varied between 9 and 15, and the b value ranged from 39 to 47. The CIE Lab* color values of the fruits were found to exhibit similar characteristics to those of apricots from the Kayseri region [14]. In this study, the sensory quality attributes of apricot samples were evaluated using easily measurable physical parameters, including external skin color, fruit weight, and size, together with sweetness-related indicators. However, comprehensive sensory attributes such as taste and aroma, as well as textural properties including fruit firmness, were not included within the scope of this study. These limitations are acknowledged, and the incorporation of detailed sensory analysis and texture-related parameters is recommended for future studies to achieve a more complete assessment of apricot sensory quality. Skin color is a key quality indicator closely linked to fruit maturity, consumer acceptance, and the accumulation of bioactive compounds.

The acidity of apricot samples was quantified through titratable acidity, expressed as malic acid equivalent. Acidity is a key quality attribute in fruits, as it plays an important role in sensory perception, fruit maturity, product stability, and the bioavailability of bioactive compounds [14,15]. The results demonstrated a wide range of titratable acidity (TA) values, spanning from 0.27% to 0.90%. The lowest acidity was recorded in the Malatya (A-6), while the highest was observed in the Amasya (A-7). Among the Nakhchivan varieties, no significant differences in TA were detected. Comparable findings were reported in a study on apricots from the Pakistan region, where titratable acidity ranged between 0.31% and 0.34% as malic acid [3]. In a study conducted on different apricots using similar analytical methods, a variation ranging from 0.90% to 2.28% was reported [15]. In a study, titratable acidity was calculated in terms of citric acid, and it was reported that the Malatya apricot variety showed the lowest TA value among the samples [3]. Similarly, in our study, the Malatya variety was found to have a notably low TA value, consistent with previous findings.

This indicates consistency across different geographical regions and highlights the variability of TA levels due to genetic and environmental factors. Titratable acidity is a critical quality parameter for apricots as it significantly influences the fruit’s sensory properties, including taste and aroma. Higher TA levels often result in a tangy flavor, while lower levels are associated with a sweeter taste profile. Furthermore, TA is widely used as an indicator of fruit maturity and ripeness, providing insights into the optimal harvesting period. Organic acids, such as ascorbic acid, citric acid, malic acid, succinic, oxalic, and phenolic acids collectively define the total acidity of fruits [16,17,18,19,20]. These compounds play an integral role in shaping the flavor, aroma, and overall quality of apricots, underscoring their significance in both consumer preferences and commercial applications [3,4].

### 2.2. Ascorbic Acid and Sugar Profiles of Apricot Extracts

**Figure 1:**
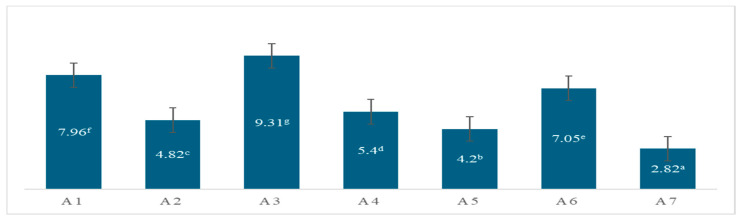
Ascorbic acid contents of the apricot samples (mg/100 g). Different letters (a–g) in the same lines are significantly different at the 5% level (*p* < 0.05).

Ascorbic acid is one of the major organic acids present in fruits, alongside citric acid, and phenolic acids, which are among the key organic compounds contributing to the fruit’s chemical composition and quality. In this study, the analysis focused on ascorbic acid and specific phenolic acids (Figure 1). The ascorbic acid content was quantified using a titrimetric method, ensuring precise and reliable measurement. This approach allowed for the simultaneous evaluation of key organic compounds that contribute to the antioxidant properties and overall quality of the fruit. The ascorbic acid content in the apricot samples was found to range from 2.82 to 9.31 mg/100 g. Among the varieties analyzed, the A-1 and A-3 varieties exhibited the highest ascorbic acid concentrations. Studies on apricots have reported that the major organic acids are citric acid and malic acid, while the ascorbic acid content is generally lower than that of these two organic acids [3,4]. In Hungary, apricot samples from different genotypes had ascorbic acid levels ranging from 3.04 to 16.17 mg/100 g FW [11].

The values in the table are given as average values. Letters in each cell indicate differences at the *p* < 0.05 (one-way ANOVA test) level.

The sugar profile was determined by HPLC-RID through the quantification of eight individual sugars, and the corresponding results are presented in Table 2. Sucrose emerged as the predominant sugar in the fruits, followed by glucose, melibiose, fructose, and trehalose. Notably, ribose, maltose and melezitose were not detected in any fruits.

Fructose, commonly referred to as fruit sugar, is a primary sugar naturally found in most fruits. However, unlike many other fruits such as figs [20], grapes [21], dates [22] and apples [22], apricots do not typically exhibit higher levels of fructose compared to sucrose and glucose [16]. This deviation is a characteristic feature of apricots, distinguishing their sugar composition profile from that of other fruits. Similarly, other studies have reported that fructose levels in apricots are lower than those of sucrose and glucose. For instance, a study on Malatya apricots reported sucrose concentrations ranging from approximately 22.96 to 56.83 mg/100 g, glucose concentrations from 9.47 to 23.67 mg/100 g, and fructose concentrations from 6.34 to 15.68 mg/100 g [3]. A study comparing local and foreign apricot varieties reported that sugar concentrations follow the descending order of sucrose, glucose, fructose, and maltose. In this study, it was shown that sucrose exhibited the greatest variation in its concentration [18]. However, the different studies also highlighted the presence of significant amounts of sorbitol in apricots, with sorbitol concentrations varying between 4.26 and 8.16 mg/g depending on the apricot variety [17]. The higher content of melibiose compared to fructose in apricots is considered a notable finding. Melibiose, a disaccharide, is formed by the α-1,6-glycosidic bond between galactose and glucose molecules. This sugar, which is typically found in high concentrations in honeydew honey, cannot be directly metabolized by the human digestive system; however, it is fermented by gut microorganisms, providing a prebiotic effect [23,24]. Another disaccharide present in apricots in relatively low amounts is trehalose, with values ranging from 0.01 to 0.48 g/100 g. The highest concentration of this sugar was observed in Malatya apricots. Trehalose, like melibiose, is considered to be sugar with prebiotic properties. This disaccharide, formed by an α,α-1,1-glycosidic bond between two glucose molecules, cannot be directly metabolized in humans due to the absence of the enzyme trehalase. Instead, trehalose is fermented and broken down by gut microorganisms, a process that provides prebiotic effects and supports gut health [25,26,27].

The differences in sugar composition among the analyzed *P. armeniaca* varieties can be attributed to environmental and agroclimatic conditions affecting carbohydrate metabolism during fruit development and ripening. Temperature, sunshine, water availability, and soil characteristics influence photosynthetic activity and carbon distribution, determining sugar synthesis and accumulation [17,19]. And, also, high glucose and fructose levels in some varieties can be explained by increased solar radiation and high temperatures enhancing photosynthesis and sucrose hydrolysis, while a lower sugar content may be attributed to restricted carbohydrate transport due to cool climate conditions, limited light, or water stress [22,23].

Different types of sugars are important for health because they affect glycemic response, energy metabolism, and nutritional quality. In fruits, the ratios of glucose, fructose, and sucrose determine taste perception and blood sugar response, while their presence along with fiber and bioactive compounds contributes to their potential as functional foods [24]. Fructose, a fruit sugar, is one of the sweetest sugars. Although the fructose content is relatively low in apricot fruits, it appears that the fruit’s sweetness primarily results from other sugars. When calculating the sweetness index (SI) of the fruit, based on the combined function of fructose, glucose, and sucrose, the highest SI was found in A-4 and A-6 apricots. It was determined that the apricot with the lowest sweetness value was the A-7 variety. While organic acids (such as citric acid, ascorbic acid, malic acid) and organic alcohols (such as sorbitol) in the fruit give the fruit its sourness, fructose, sucrose and some oligosaccharides give it its sweetness [1,17]. Indeed, when the relationship between acidity and sweetness index was examined using Pearson correlation, a negative correlation was found between them (r = −0.712, *p* < 0.01). A similar relationship has also been demonstrated in other studies [17,19]. The sugar composition determines the SI, which is a measure of the sweetness value of apricot fruit. The relationship between the measured sugar values and SI indices is provided in Table 3, using Pearson correlation.

### 2.3. Antioxidant Activity of Apricot Extracts

The total phenolic content (TPC) and antioxidant capacities of methanolic apricot extracts are given in Table 4. The TPC values were found to range widely, from 10.04 mg GAE/100 g FW to 85.88 mg GAE/100 g FW. Similarly, the total flavonoid content varied between 0.21 mg QE/100 g FW and 3.36 mg QE/100 g FW. The highest total phenolic content was observed in the A-2 and A-7 varieties, whereas it was lower in the A-3 and A-5 apricot varieties. Similarly, the total flavonoid content was also found to be highest in A-2 and A-7, consistent with the trends observed for total phenolic content.

Table 5 presents some phenolic properties reported in the literature for different apricot varieties. Previous studies have shown that apricots are rich in various phenolic compounds, primarily phenolic acids and flavonoids, but the quantity and distribution of these compounds vary significantly depending on the variety [25]. These differences are reported to stem from factors such as genetic structure, growing conditions, and maturity level. In particular, compounds such as chlorogenic acid, caffeic acid, and rutin stand out as dominant phenolics in apricots and make a significant contribution to their antioxidant potential. A study of 27 apricot genotypes reported that total phenolic content ranged from 82.0 to 2891.6 mg GAE/L and 168.5 to 3499.6 mg GAE/L in 2006 and 2007, respectively [8]. Another apricot study reported that total phenolic amounts ranged from 29 to 69 mg GA/100 g. These findings demonstrate that apricot represents a nutritionally valuable fruit with significant potential health benefits [28].

The antioxidant activity of the methanolic extracts was assessed using two distinct methods (Table 4). The Ferric Reducing Antioxidant Power (FRAP) assay, a widely recognized and straightforward method, measures antioxidant capacity based on the reduction of the Fe^3+^- TPTZ complex to its ferrous form (Fe^2+^) in the presence of antioxidants. Higher FRAP values correspond to a greater antioxidant potential. The FRAP values of the samples exhibited a wide range, from 9.08 mg TE/100 g FW to 81.84 mg TE/100 g FW, with the A-7 and A-2 samples demonstrating the highest antioxidant capacity. Similarly, the DPPH^•^ radical scavenging activity assay, which evaluates the ability of antioxidants to neutralize free radicals, revealed a significant relationship between SC_50_ values and radical inhibition efficacy. Lower SC_50_ values indicate a stronger radical scavenging effect. Among the samples analyzed, A-7 and A-2 displayed the highest radical scavenging activity. A robust negative correlation was observed between the FRAP values and DPPH^•^ radical scavenging activities (r = −0.832, *p* < 0.05), underscoring the consistency of these methods in assessing antioxidant potential. The DPPH assay was chosen as a widely used and well-established method for assessing the free radical scavenging capacity of natural products. Its simplicity and reproducibility allow reliable comparison with previous studies on fruit- and plant-derived extracts. Together with ferric reducing power, the DPPH assay was considered adequate to represent the antioxidant potential of the apricot extracts within the scope of this study [29,30].

**Table 5:** Biochemical properties of selected apricot cultivars reported in the literature.

References	Total Phenolic Content (TPC)	Total Flavonoid Content (TFC)	Total Antioxidant Capacity (FRAP)	Major Phenolics	Cited
32 cultivars of different origin	63.5–1277.30 mg GAE/100 g DW	0–153.10 mg CE/100 g DW	483.4–2348.40 mg TE/100 g DW	-	[31]
11 apricot genotypes	24.87–41.31 (GAE/g)	-	-	-	[32]
14 apricot genotypes	92.20–162.10 (mg GAE/100 g)	-	154.10–243.60 FeSO4.7H_2_O μg/mL	-	[33]
	41–170 mg GAE/100 g FW	-	12.00–102.90 mg TE/100 g FW	chlorogenic acid, salicylic acid, quercetin, rutin	[34]
27 apricot cultivars and hybrids of diverse origins	82.0–2891.60 mg GA/L	-	0.48 to 14 mmol AA/L	-	[11]
10 apricot genotypes	69.3–80.80 mg GAE/100 g	9.20–15.10 mg CE/100 g	-	-	[13]
14 apricot varieties	-	24.2–72.50 mg/100 g	12.2–36.10 mg/100 g	*m*-coumaric acid, chlorogenic acid	[35]

Antioxidant activity was evaluated to determine the capacity of the fruit to counteract oxidative stress through its bioactive compounds. This parameter is particularly important for functional foods, as it reflects potential health-promoting effects beyond basic nutrition and supports the biological relevance of the obtained results. The antioxidant capacity of apricots is primarily attributed to their content of phenolic compounds and vitamins, including ascorbic acid and β-carotene [3,32]. The strong correlations observed between total phenolic content (TPC), total flavonoid content (TFC) and the results of FRAP and DPPH^•^ assays further substantiate this relationship (Table 5). These findings highlight the significant contribution of phenolic substances and vitamins to the overall antioxidant activity of apricots. A study examining 27 distinct apricot species identified genotypes as the primary determinant of antioxidant capacity. Researchers have identified that genotypes are the most significant determining factor for variations in antioxidant capacity, although the harvest year also exerts a notable effect on total phenolic content [11,32].

### 2.4. Phenolic Compounds of Apricot Extracts

Chromatographic profiles of phenolic components in apricot methanol extracts were determined using the RP-HPLC-PDA method, and the results of analyses performed according to 25 phenolic standards are presented in Table 6. The findings revealed that all apricot varieties examined generally exhibited similar profiles in terms of phenolic composition, but there were significant differences between varieties in the concentrations of individual phenolic compounds. The phenolic standards used for calibration were selected to represent the major polyphenolic compounds commonly reported in natural products. Care was taken to include compounds that showed good chromatographic separation and no peak overlap or interference in HPLC-PDA analysis, thereby ensuring reliable and reproducible quantification [36]. Among the phenolic standards used in the study, only four compounds were consistently detected in all apricot samples: chrysanthemum, pinosembrin, galangin, and *t*-cinnamic acid. Other phenolic components were not evaluated as they fell below the LOD and LOQ values (Table S1, Figure S1). The common presence of these compounds in all samples suggests that this quadruple phenolic compound pattern could be considered a distinctive phenolic profile or a potential “phenolic fingerprint” for apricot samples.

A study conducted on domestic and foreign apricot varieties in Türkiye detected the presence of chlorogenic acid, caffeic acid, syringic acid, *p*-coumaric acid, ferulic acid, protocatechuic acid, vanillic acid, and gallic acid. However, in the current analysis, these phenolic compounds were found below detectable levels in the samples [19]. A study like ours has also demonstrated that cinnamic acid, chrysin, and pinocembrin are among the major phenolic compounds found in strawberries [29]. HPLC analysis of fourteen apricot varieties originating from North India revealed a phenolic profile that differs from the findings of the present study [28]. Similarly, another investigation conducted on local apricot cultivars reported chlorogenic acid, protocatechuic acid, caffeic acid, ferulic acid, and gallic acid as the predominant phenolic compounds [18]. These discrepancies in phenolic composition may be attributed to inter-cultivar variability as well as to methodological differences, particularly variations in the extraction procedures employed, which are known to significantly influence the qualitative and quantitative recovery of phenolic compounds. Phenolic compounds are of interest in functional foods due to their strong antioxidant, anti-inflammatory properties, as well as potential protective effects against oxidative stress-related diseases. Their presence enhances the health-promoting value of food beyond basic nutrition [29,37]. A total of 25 phenolic standards were screened using HPLC-PDA analysis to determine the phenolic composition; however, within the detection and quantification limits of the method, only chrysin, pinocembrin, galangin, and cinnamic acid could be detected. The limited number of compounds in the phenolic profile may be related to the lower sensitivity of the HPLC-PDA method compared to more advanced analytical techniques with higher sensitivity and selectivity, such as LC-MS/MS or GC-MS. Therefore, the phenolic compounds identified in this study reflect those that could be determined under the applied analytical conditions.

### 2.5. Principal Component Analysis (PCA)

**Figure 2:**
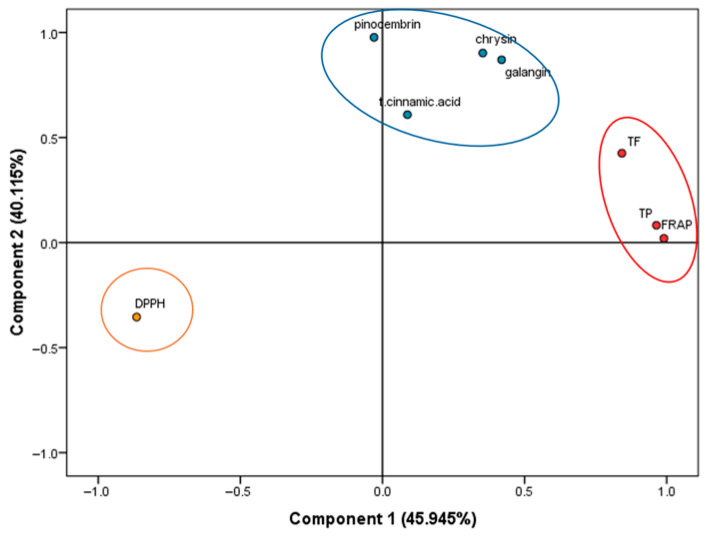
PCA score plot is based on the TPC, TFC, DPPH^•^, FRAP and phenolic acid parameters.

Before applying PCA, all variables were standardized using autoscaling (mean centering and division by standard deviation) to prevent bias that might arise from different units of measurement. The clustering observed in the PCA score graph was found to be directly related to differences in the biochemical profiles of the varieties, particularly changes in phenolic compound content and antioxidant-related parameters. Variance explanation ratios exceeding 70% are generally considered sufficient to ensure the reliability and interpretability of PCA models [38]. In the present study, the first two principal components (PC1 and PC2) together accounted for 86.059% of the total variance, with PC1 contributing 45.945% and PC2 contributing 40.115% (Figure 2). This high cumulative variance indicates that the multidimensional structure of the dataset is effectively captured within a two-component model.

In accordance with Kaiser’s criterion, which designates components with eigenvalues greater than 1.0 as meaningful descriptors of data structure [38,39], both PC1 (4.932) and PC2 (1.952) exceeded this threshold. The magnitude of these eigenvalues suggests that each component encapsulates a substantial portion of the dataset’s informational content and contributes significantly to the overall discriminatory power of the model.

Collectively, these results confirm the robustness and statistical adequacy of the PCA. The combination of a high cumulative explained variance and eigenvalues surpassing the Kaiser threshold indicates that PC1 and PC2 provide a comprehensive and reliable representation of the underlying relationships among the variables. Thus, the principal components derived in this analysis can be considered effective in summarizing the dominant patterns and driving sources of variability within the dataset.

## 3. Materials and Methods

### 3.1. Apricot Samples and Extraction

In June 2024, a minimum of 1 kg of ripe apricot samples was collected from gardens (Figure S3). The samples were placed in polyethylene bags and transported to the laboratory under cold-chain conditions. The average length and weight of each apricot variety were determined using measurements from at least 20 individual apricots. Fresh samples were stored at 4 °C until analysis, with a maximum storage period of five days. Depending on the specific analysis parameters, either aqueous or methanolic extraction methods were employed. Table 7 shows the codes, genotypes, and growing regions of the apricot varieties used in the study.

Aqueous apricot extracts were prepared using distilled water as the solvent. Each apricot sample was homogenized with distilled water and subsequently filtered. The obtained filtration was then centrifuged at 1000 rpm for 15 min to remove particulate matter and obtain a clear extract. The methanolic apricot extract was prepared using 80% methanol as the extraction solvent. A total of 100 g of apricot mixture was homogenized with 250 mL of 80% methanol and subjected to ultrasonic-assisted extraction for 2 h, followed by agitation in a shaker for 6 h. The resulting extract was filtered and stored in aliquots at deep-freeze temperatures until further analysis [37].

### 3.2. Determination of Physicochemical Characteristics

The dry matter content was determined in accordance with AOAC protocols [10]; soluble solid content, expressed as a percentage (%), was measured in the juice of each sample using an Abbe-3 L refractometer (Tokyo, Japan) at 20 °C.

Following centrifugation of the aqueous apricot extract, the supernatant was filtered and subsequently diluted at a ratio of 1:20 with bidistilled water. An aliquot of the diluted filtrate was then titrated with 0.01 M NaOH using phenolphthalein as an indicator. The titratable acidity was calculated and expressed as percentage of malic acid equivalents, in accordance with the method described by Wani et al. [40]. The aqueous extracts were prepared for the determination of titratable acidity (TA). TA was analyzed by titrating 5 mL of juice with 0.1 M NaOH, and the results were reported as a percentage of malic acid, following the guidelines outlined in AOAC [10]. All analyses were performed in triplicate to ensure the reliability and reproducibility of the results. The color of fresh apricot samples was measured using the classical method for food analysis, based on the CIE Lab* color space model (Konica Minolta, CR-400 Plus, Tokyo, Japan). In this model: L* represents lightness, with values ranging from 0 (black) to 100 (white); a* denotes the color axis from green (−a) to red (+a); and b* represents the color axis from blue (−b) to yellow (+b).

### 3.3. Determination of Ascorbic Acid Amount

The amount of ascorbic acid in aqueous solution was measured through classical titrimetric analysis based on the redox reaction between Potassium iodate (KIO_3_) and ascorbic acid. It uses the principle of oxidation of ascorbic acid by potassium iodate, a strong reducing agent [41]. The experiment utilized the reaction of iodate ions with iodide ions in an acidic solution to generate iodine. For this purpose, KI (1 g), HCl (1 M, 5 mL), and starch solution were added to the sample, which had been diluted at a ratio of 1:50 to contain vitamin C (Sigma-Aldrich, Merck, Darmstadt, Germany). The mixture was then titrated with 5 × 10^−5^ M KIO_3_. Calculations based on the reaction were performed to determine the ascorbic acid content, expressed as mg/100 g FW.

### 3.4. Determination of Sugar Profiles

For the sugar profile analysis, an Elite LaChrom Hitachi HPLC system equipped with a refractive index detector (RID) was utilized. The analysis employed eight sugar standards: ribose, fructose, glucose, sucrose, maltose, trehalose, melibiose, and melezitose. All analytical standards (≥98% purity) were purchased from Sigma-Aldrich, Merck (Darmstadt, Germany). Standard solutions at varying concentrations (40, 20, 10, 5, 2.5, and 1.25 mg/mL) were prepared, filtered through a 0.45 μm membrane, transferred to vials, and loaded onto the HPLC autosampler. Calibration curves were constructed from the resulting data prior to analysis.

For sample preparation, 1 g of homogenized apricot was mixed with 10 mL of 80% ethanol and filtered through filter paper, and the filtrate was evaporated to dryness. The residue was reconstituted in ultrapure water, filtered through a 0.45 μm membrane, and injected into the HPLC. A reverse-phase -NH_2_ column (EC 250/4 Nucleosil, Macherey-Nagel, Düren, Germany) was employed for separation, using an isocratic mobile phase of 80% acetonitrile and 20% ultrapure water. The injection volume for both standards and samples were optimized at 20 μL, with a mobile phase flow rate of 1.5 mL/min and a column temperature of 30 °C [42].

### 3.5. Determination of Sweetness Index (SI)

The sweetness index (SI) of the fruit, which serves as an estimate of the overall sweetness perception, was calculated by considering the relative amounts and sweetness properties of individual carbohydrates [1]. The contribution of each carbohydrate to the overall sweetness was determined based on the relative sweetness values: fructose is 2.3 times sweeter than glucose; sucrose is 1.35 times sweeter; and sorbitol is 0.81 times sweeter than glucose. Consequently, the SI was calculated using Formula (1).

SI = (1.00 × glucose) + (1.35 × sucrose) + (2.3 × fructose),(1)

This method enables the quantification of sweetness based on the contribution of each sugar, providing a more comprehensive measure of the fruit’s overall sweetness profile; however, since sorbitol was not included in the analysis, its contribution to the sweetness was not considered.

### 3.6. Determination of Total Phenolic Contents (TPC)

The total phenolic content was determined spectrophotometrically using the Folin–Ciocalteu method [43]. In this method, methanolic apricot extracts were used. A reaction mixture was prepared by combining 50 µL of the extract with 400 µL of 0.2 N Folin–Ciocalteu reagent, followed by dilution with 680 µL of distilled water. After a 3 min incubation at room temperature, 400 µL of 10% Na_2_CO_3_ was added, and the mixture was incubated at 25 °C for 2 h. The absorbance of the reaction was measured at 760 nm using a UV-Vis spectrophotometer (Thermo Scientific Evolution TM 201, Madison, WI, USA). A calibration curve was generated using six gallic acid standards ranging from 0.0625 to 1.00 mg GAE/100 mL. The total phenolic content was expressed as mg GAE/100 g FW, based on the standard curve.

### 3.7. Determination of Total Flavonoid Contents (TFC)

The total flavonoid content was determined in the methanolic extract using a modified method based on Fukumoto and Mazza [44]. In this modification, AlNO_3_ was used in place of AlCl_3_ due to its lower solubility in water. To begin, 50 µL of ethanolic extract was combined with 50 µL of 10% Al (NO_3_)_3_ and 50 µL of 1.0 M NH_4_CH_3_COO. The mixture was then diluted to a final volume of 3.0 mL with 99% ethanol and incubated at 25 °C for 45 min. After incubation, the absorbance was measured at 415 nm. A calibration curve was constructed using six different quercetin standards, ranging from 0.031 to 0.50 mg QE/100 mL. The total flavonoid content was expressed as mg QE/100 g FW, based on the standard curve.

### 3.8. Determination of Ferric Reducing Antioxidant Power (FRAP)

The total antioxidant activity of the methanolic extract was assessed using the Ferric-Reducing Antioxidant Power (FRAP) assay [45]. To prepare the FRAP reagent, ferric tripyridyl-triazine (Fe-III-TPTZ) (Sigma-Aldrich, Darmstadt, Germany), acetate buffer in 40 mM HCl, and a 2.5 mL solution of mM FeCl_3_.6H_2_O were combined in a test tube. Subsequently, 3 mL of the FRAP reagent was mixed with 100 µL of the extract and incubated for 4 min at 37 °C. The absorbance was measured at 595 nm. A standard calibration curve was generated using different concentrations of FeSO_4_.7H_2_O (ranging from 1000 to 31.25 µmol/mL). The results were reported as µmol FeSO_4_.7H_2_O/100 g FW.

### 3.9. Determination of DPPH^•^ Radical Scavenging Capacity

The DPPH^•^ radical scavenging activity was evaluated using a spectrophotometric method [46]. In this assay, 1.0 mL of 0.04 mg/mL DPPH^•^ radical (Sigma-Aldrich, Darmstadt, Germany) solution was mixed with 1.0 mL of the extract. The mixture was then kept in the dark for 45 min at 25 °C, after which the absorbance was measured at 517 nm. To determine the SC_50_ value, six different dilutions of the ethanolic extract were prepared and treated with the DPPH^•^ solution, followed by absorbance measurement at 517 nm. The SC_50_ value, representing the concentration of extract required to scavenge 50% of the DPPH^•^ radical, was obtained from the resulting graph.

### 3.10. Determination of Phenolic Composition Using HPLC-PDA

Prior to the phenolic component analysis of methanolic extracts via HPLC–PDA, liquid–liquid extraction was conducted for enrichment. A 10 mL aliquot of the extract was evaporated at 40 °C using a rotary evaporator. The residue was dissolved in 10 mL of distilled water, and the pH was adjusted to 2 using concentrated HCl. The organic phases were combined after three successive extractions with diethyl ether and ethyl acetate. Following solvent removal, the residue was dissolved in 2 mL of methanol, filtered through a 0.45 μm RC membrane, and subjected to phenolic analysis.

The phenolic composition of the mixture was analyzed using an HPLC-PDA system equipped with a photodiode array (PDA) detector (Shimadzu Liquid Corporation LC 20AT, Kyoto, Japan) and a C18 column (250 mm × 4.6 mm, 5 µm; GL Sciences, Tokyo, Japan). A calibration curve was constructed using 25 phenolic standards. The mobile phase consisted of (A) 2% acetic acid in water and (B) a mixture of acetonitrile and water (70:30). Sample and standard injection volumes were set at 20 µL, with a column temperature of 30 °C and a flow rate of 1.0 mL/min [36]. In addition, analytical validation parameters for the phenolic compounds, including the limits of detection (LOD) and limits of quantification (LOQ), as well as linearity ranges and correlation coefficients, were thoroughly established and reported in our previous study [36].

### 3.11. Statistical Analysis

All experiments were conducted in triplicate, with results expressed as mean ± standard deviation. Data analysis was performed using SPSS software (version 21; IBM Corp., Armonk, NY, USA, 2012). One-way ANOVA test was used to find differences. The correlations between sugar components and antioxidant parameters were analyzed by Pearson correlation. Principal component analysis was also performed using antioxidants and phenolic components. *p* < 0.01 and *p* < 0.05 were used as statistical significance levels.

## 4. Conclusions

The present study comprehensively evaluated the physicochemical, biochemical, and nutritional characteristics of apricot genotypes cultivated in the Nakhchivan region of Azerbaijan and in Türkiye, revealing substantial inter-genotypic and inter-regional variability. Fruit weight, color parameters (Hunter L* and a*), soluble solid content, and titratable acidity (TA) exhibited wide ranges, reflecting significant differences in pomological and quality-related traits. According to the CIE L*a*b* color system, the apricot samples exhibited L* (lightness) values ranging from 61.82 to 72.58, a* (greenness) values from 13.83 to 26.91, and b* (yellowness) values from 45.63 to 52.58. Although variations were observed in the numerical color parameters, all samples were visually characterized by a light-yellow appearance, indicating a generally similar overall color profile despite measurable differences. The ascorbic acid concentrations in the analyzed samples were found to range from 2.82 to 9.31 mg/100 g, indicating a significant difference in content between the samples. In the analyzed samples, sucrose and glucose were identified as the dominant sugars, while fructose levels were found to be low. The total phenolic content (TPC) in the analyzed samples ranged from 10.04 to 85.88 mg GAE/g. The highest TPC values were detected in the Nakhchivan Adi shalax (A2) and Amasya (A7) samples, respectively. In addition, a positive correlation was observed between TPC and antioxidant capacity measurements (FRAP and DPPH), supporting the contribution of phenolic compounds to antioxidant activity. Apricot samples were found to contain varying concentrations of the same polyphenolic compounds, including chrysin, pinocembrin, galangin, and *trans*-cinnamic acid, indicating qualitative consistency but quantitative variability in their phenolic profiles.

Overall, the observed genotype–environment interactions appear to shape the biochemical profile of apricots, and certain cultivars demonstrated more favorable compositional characteristics, suggesting their potential suitability for functional food and nutraceutical applications. Nevertheless, further in vivo and clinical investigations are required to substantiate specific health-related claims.

## Figures and Tables

**Table 1:** Physicochemical characteristics of the apricot fruits.

Apricot Fruits	A-1	A-2	A-3	A-4	A-5	A-6	A-7
Weight (g)	74.10 ± 6.65 ^d^	58.97 ± 1.80 ^b.c^	60.33 ± 2.40 ^b.c^	42.03 ± 3.38 ^a^	55.34 ± 4.26 ^a.b^	42.56 ± 2.50 ^a^	70.40 ± 8.42 ^c.d^
Seedless weight (g)	72.96 ± 5.00 ^c^	54.89 ± 6.30 ^b^	55.44 ± 2.28 ^b^	40.21 ± 1.95 ^a^	49.96 ± 3.37 ^a.b^	40.58 ± 0.94 ^a^	71.56 ± 3.53 ^c^
Length (mm)	57.00 ± 6.25 ^c^	50.67 ± 2.52 ^b.c^	53.00 ± 2.65 ^b.c^	41.33 ± 2.52 ^a^	51.33 ± 2.52 ^b.c^	47.00 ± 1.00 ^a.b^	51.67 ± 5.46 ^b.c^
Width (mm)	50.33 ± 3.22 ^a.c^	45.67 ± 0.58 ^a.b.c^	48.00 ± 2.00 ^b.c^	42.67 ± 1.53 ^a.b^	44.00 ± 2.00 ^a.b.c^	41.00 ± 2.65 ^a^	50.33 ± 3.51 ^a.c^
Seed weight (g)	2.81 ± 0.30 ^a.b^	2.23 ± 0.18 ^a^	2.74 ± 0.24 ^a^	3.39 ± 0.17 ^b^	2.39 ± 0.22 ^a^	2.84 ± 0.10 ^a.b^	2.76 ± 0.31 ^a^
Soluble solid %	18.52 ± 0.20 ^c^	19.80 ± 0.35 ^d^	19.44 ± 0.23 ^c.d^	18.72 ± 0.24 ^c^	13.47 ± 0.20 ^a^	20.86 ± 0.77 ^e^	16.15 ± 0.31 ^b^
Titratable acidity (Malic acid%)	0.41 ± 0.03 ^b^	0.46 ± 0.02 ^b^	0.55 ± 0.02 ^c^	0.43 ± 0.02 ^b^	0.46 ± 0.02 ^b^	0.27 ± 0.02 ^a^	0.90 ± 0.07 ^d^
(CIE Lab*) L	67.82 ± 3.86 ^a.b^	72.82 ± 1.27 ^b.c^	72.04 ± 3.24 ^b.c^	72.58 ± 1.52 ^b.c^	75.47 ± 1.28 ^c^	71.15 ± 2.32 ^b.c^	61.82 ± 3.03 ^a^
(CIE Lab*) a	23.68 ± 2.03 ^b.c^	21.78 ± 0.88 ^b^	22.04 ± 2.41 ^b^	13.83 ± 1.66 ^a^	21.00 ± 1.23 ^b^	19.65 ± 2.20 ^b^	26.91 ± 0.80 ^c^
(CIE Lab*) b	47.76 ± 2.30 ^a^	50.27 ± 3.09 ^a^	47.66 ± 3.60 ^a^	52.58 ± 1.70 ^a^	47.96 ± 2.49 ^a^	49.52 ± 3.84 ^a^	45.63 ± 3.84 ^a^

The values in the table are given as average values. Letters in each cell indicate differences at *p* < 0.05 (one-way ANOVA test) level.

**Table 2:** Sugar profiles and sweetness index (SI) of the apricot fruit samples.

(g/100 g FW)	A-1	A-2	A-3	A-4	A-5	A-6	A-7
Ribose	-	-	-	-	-	-	-
Fructose	0.67 ± 0.02 ^c^	0.66 ± 0.03 ^c^	1.08 ± 0.06 ^d^	0.03 ± 0.01 ^a^	-	0.26 ± 0.03 ^b^	-
Glucose	4.34 ± 0.03 ^c^	5.39 ± 0.04 ^e^	4.29 ± 0.03 ^c^	4.12 ± 0.04 ^b^	4.75 ± 0.03 ^d^	8.77 ± 0.04 ^f^	1.10 ± 0.01 ^a^
Sucrose	12.81 ± 0.19 ^b^	12.44 ± 0.21 ^b^	12.17 ± 0.54 ^b^	19.30 ± 0.27 ^c^	8.48 ± 0.32 ^a^	9.31 ± 0.21 ^a^	12.66 ± 0.48 ^b^
Maltose	-	-	-	-	-	-	-
Trehalose	0.14 ± 0.02 ^a,c^	0.03 ± 0.03 ^a,b^	0.07 ± 0.02 ^a,b,c^	0.10 ± 0.04 ^b,c^	0.01 ± 0.01 ^a^	0.48 ± 0.05 ^d^	0.03 ± 0.01 ^a,b^
Melibiose	1.00 ± 0.05 ^c^	0.92 ± 0.04 ^c^	1.71 ± 0.06 ^e^	0.54 ± 0.05 ^a^	3.05 ± 0.06 ^f^	0.73 ± 0.06 ^b^	1.30 ± 0.07 ^d^
Melezitose	-	-	-	-	-	-	-
Total sugar	18.99 ± 0.03 ^c^	19.45 ± 0.03 ^d^	19.31 ± 0.14 ^d^	23.91 ± 0.04 ^e^	16.36 ± 0.05 ^b^	19.51 ± 0.04 ^d^	14.99 ± 0.13 ^a^
Sweetness index	27.99 ± 0.13 ^c^	29.88 ± 0.08 ^d^	27.37 ± 0.79 ^c^	35.32 ± 0.18 ^f^	22.46 ± 0.15 ^b^	32.95 ± 0.10 ^e^	19.49 ± 0.74 ^a^

(-) not detected. The values in the table are given as average values. Letters in each cell indicate differences at *p* < 0.05 (one-way ANOVA test) level.

**Table 3:** Pearson correlation coefficients among individual sugar components of the apricot fruits.

	Fru	Glu	Suc	Tre	Mel	TS	SI
Fru	1	0.150	−0.074	−0.038	−0.111	0.152	0.162
Glu		1	−0.373	0.777 **	−0.183	0.358	0.634 **
Suc			1	−0.237	−0.590 **	0.703 **	0.475 *
Tre				1	−0.449 *	0.240	0.519 *
Mel					1	−0.599 **	−0.668 **
TS						1	0.932 **
SI							1

**: *p* < 0.01, *: *p* < 0.05, Fru: fructose, Glu: glucose, Suc: sucrose, Tre: trehalose, Mel: melibiose, TS: Total Sugar, SI: sweetness index.

**Table 4:** Total phenolic and flavonoid content, and antioxidant capacities of the apricot fruits.

	A-1	A-2	A-3	A-4	A-5	A-6	A-7
Total Phenolic Content (mg GAE/100 g FW)	25.48 ± 0.69 ^c^	85.88 ± 3.96 ^d^	10.28 ± 2.26 ^a^	27.83 ± 1.68 ^c^	10.04 ± 0.34 ^a^	16.59 ± 0.33 ^b^	85.32 ± 1.38 ^d^
Total Flavonoid Content (mg QE/100 g FW)	1.75 ± 0.08 ^e^	3.36 ± 0.13 ^f^	1.10 ± 0.03 ^c^	0.64 ± 0.10 ^b^	0.21 ± 0.02 ^a^	1.37 ± 0.08 ^d^	1.97 ± 0.13 ^e^
FRAP (µmol FeSO_4_.7H_2_O/100 g FW)	19.53 ± 0.10 ^d^	77.91 ± 1.14 ^e^	11.29 ± 0.65 ^b^	10.12 ± 0.97 ^a,b^	9.08 ± 0.17 ^a^	16.35 ± 0.30 ^c^	81.84 ± 0.28 ^f^
DPPH^•^ radical scavenging activity SC_50_ (mg/mL)	25.06 ± 0.53 ^b^	7.18 ± 0.37 ^a^	56.28 ± 1.58 ^d^	74.45 ± 1.91 ^e^	77.02 ± 2.00 ^e^	34.72 ± 1.74 ^c^	6.32 ± 0.19 ^a^

Different letters (a–f) in the same lines are significantly different at the 5% level (*p* < 0.05).

**Table 6:** Phenolics profiles of the apricot samples from 25 phenolic compounds from HPLC-PDA.

	A-1	A-2	A-3	A-4	A-5	A-6	A-7
Phenolic acids (µg/100 g FW)							
*t*-Cinnamic acid	48.60	56.60	19.80	13.80	42.50	13.50	5.50
Flavonoids (µg/100 g FW)							
Chyrisin	68.10	61.20	39.90	52.10	34.80	46.4	49.2
Pinocembrin	67.20	46.60	38.70	43.90	28.4	38.2	35.0
Galangin	64.50	54.20	38.30	44.70	35.50	40.80	50.80

Gallic acid, protocatechuic acid, p-hydroxybenzoic acid, chlorogenic acid, caffeic acid, syringic acid, vanillic acid, p-coumaric acid, ferulic acid, ellagic acid, epicatechin, rutin, myricetin, daidzein, luteolin, quercetin, resveratrol, apigenin, hesperetin, rhamnetin, and CAPE were all below the limit of detection (LOD) in the analyzed apricot samples.

**Table 7:** Apricot (*P. armeniaca*) samples and their corresponding production regions.

A1	A2	A3	A4	A5	A6	A7
Nakhchivan Şeftali şalax	Nakhchivan Adi şalax	Nakhchivan Badami	Nakhchivan Balyarim	Türkiye Iğdır	Türkiye Malatya (Hacıoğlu)	Türkiye Amasya

## Data Availability

Data will be made available upon reasonable request.

## Supplementary Materials

The following supporting information can be downloaded at: https://www.mdpi.com/article/10.3390/molecules31060988/s1.

## Abbreviations

The following abbreviations are used in this manuscript:

ANNOVA	One-way analysis of variance
DPPH^●^	2,2-diphenyl-1-picrylhydrazyl
FRAP	Ferric reducing antioxidant power
FW	Fresh weight
GAE	Gallic acid equivalents
HPLC	High-pressure liquid chromatography
PDA	Photodiode array
QE	Quercetin equivalent
RID	Refractive index detector
SI	Sweetness index
SS	Soluble solid
TA	Titratable acidity
TFC	Total flavonoid content
TPC	Total phenolic content

## References

[1] Roussos P.A., Sefferou V., Denaxa N.K., Tsantili E., Stathis V. (2011). Apricot (*Prunus armeniaca* L.) fruit quality attributes and phytochemicals under different crop load. Sci. Hortic..

[2] Zhebentyayeva T., Ledbetter C., Burgos L., Llácer G., Badenes M.L., Byrne D.H. (2012). Apricot. Fruit Breeding.

[3] Akin E.B., Karabulut I., Topcu A. (2008). Some compositional properties of main Malatya apricot (*Prunus armeniaca* L.) varieties. Food Chem..

[4] Ali S., Masud T., Abbasi K.S., Mahmood T., Hussain A. (2015). Apricot: Nutritional potentials and health benefits—A review. Ann. Food Sci. Technol..

[5] Alpaslan M., Hayta M. (2006). Apricot kernel: Physical and chemical properties. J. Am. Oil Chem. Soc..

[6] Ercisli S. (2009). Apricot culture in Türkiye. Sci. Res. Essays.

[7] Rakida A. (2023). Analysis of morphological and pomological features of apricot in the Nakhchivan Autonomous Republic of Azerbaijan. Turk. J. Agric. For..

[8] Reale A., Di Renzo T., Russo A., Niro S., Ottombrino A., Pellicano M.P. (2020). Production of low-calorie apricot nectar sweetened with stevia: Impact on qualitative, sensory, and nutritional profiles. Food Sci. Nutr..

[9] Gupta S., Chhajed M., Arora S., Thakur G., Gupta R. (2018). Medicinal Value of Apricot: A Review. Indian J. Pharm. Sci..

[10] Cunniff P. (1995). Official Methods of Analysis of AOAC International.

[11] Hegedus A., Engel R., Abrankó L., Balogh E., Blázovics A., Hermán R., Halász J., Ercisli S., Pedryc A., Stefanovits-Bányai É. (2010). Antioxidant and antiradical capacities in apricot (*Prunus armeniaca* L.) fruits: Variations from genotypes, years, and analytical methods. J. Food Sci..

[12] Akhone M.A., Bains A., Tosif M.M., Chawla P., Fogarasi M., Fogarasi S. (2022). Apricot kernel: Bioactivity, characterization, applications, and health attributes. Foods.

[13] Karatas N. (2022). Evaluation of nutritional content in wild apricot fruits for sustainable apricot production. Sustainability.

[14] Yaman M., Turan S. (2021). Determination of fruit and leaf characteristics of some apricot varieties in Kayseri ecology. Erzincan Univ. J. Sci. Technol..

[15] Lobit P., Soing P., Génard M., Habib R. (2002). Theoretical analysis of relationships between composition, pH, and titratable acidity of peach fruit. J. Plant Nutr..

[16] Ayour J., Alahyane A., Harrak H., Neffa M., Taourirte M., Benichou M. (2021). Assessment of nutritional, technological, and commercial apricot quality criteria of the Moroccan cultivar “Maoui” compared to introduced Spanish cultivars “Canino” and “Delpatriarca”. J. Food Qual..

[17] Su C., Zheng X., Zhang D., Chen Y., Xiao J., He Y., Wang B., Shi X. (2020). Investigation of sugars, organic acids, phenolic compounds, antioxidant activity, and the aroma fingerprint of small white apricots grown in Xinjiang. J. Food Sci..

[18] Etienne A., Génard M., Lobit P., Mbeguié-A-Mbéguié D., Bugaud C. (2013). What controls fleshy fruit acidity? A review of malate and citrate accumulation in fruit cells. J. Exp. Bot..

[19] Muradoglu F., Kayakeser U. (2022). Multivariate analysis of Turkish and foreign apricot cultivars based on biochemical components. Erwerbs-Obstbau.

[20] Slatnar A., Klancar U., Stampar F., Veberic R. (2011). Effect of drying of figs (*Ficus carica* L.) on the contents of sugars, organic acids, and phenolic compounds. J. Agric. Food Chem..

[21] Eyduran S.P., Akin M., Ercisli S., Eyduran E., Maghradze D. (2015). Sugars, organic acids, and phenolic compounds of ancient grape cultivars (*Vitis vinifera* L.) from Igdir province of Eastern Türkiye. Biol. Res..

[22] Assirey E.A.R. (2015). Nutritional composition of fruit of ten date palm (*Phoenix dactylifera* L.) cultivars grown in Saudi Arabia. J. Taibah Univ. Sci..

[23] Soleymani A., Shahrajabian M.H. (2017). Effects of planting dates and row distance on sugar content, root yield and solar radiation absorption in sugar beet at different plant densities. Rom. Agric. Res.

[24] Ryu J.H., Yim J.E., Suk W.H., Lee H., Ahn H., Kim Y.S., Choue R. (2012). Sugar composition and glycemic indices of frequently consumed fruits in Korea. Korean J. Nutr..

[25] Zupan A., Mikulic-Petkovsek M., Stampar F., Veberic R. (2016). Sugar and phenol content in apple with or without watercore. J. Sci. Food Agric..

[26] Seeburger V.C., D’Alvise P., Shaaban B., Schweikert K., Lohaus G., Schroeder A., Hasselmann M. (2020). The trisaccharide melezitose impacts honey bees and their intestinal microbiota. PLoS ONE.

[27] Recklies K., Peukert C., Kölling-Speer I., Speer K. (2021). Differentiation of honeydew honeys from blossom honeys and according to their botanical origin by electrical conductivity and phenolic and sugar spectra. J. Agric. Food Chem..

[28] Saridaş M., Ağçam E., Paydaş Kargı S. (2023). The nutritional composition of key apricot varieties cultivated in Türkiye with a focus on health-related compounds. Int. J. Agric. Environ. Food Sci..

[29] Dinç S., Kara M., Takma Ç., Kara Y., Kolaylı S. (2024). Strawberries from Konya in the Central Anatolia Region of Türkiye: Phenolic profile, antioxidant potential, and mineral composition. Appl. Fruit Sci..

[30] Kolaylı S., Tunca E., Özkök A. (2025). The Botanic, physicochemical, phenolic and antioxidant properties of Muş Basin honeys. J. Apitherapy Nat..

[31] Rampáčková E., Göttingerová M., Gála P., Kiss T., Ercişli S., Nečas T. (2021). Evaluation of protein and antioxidant content in apricot kernels as a sustainable additional source of nutrition. Sustainability.

[32] Wani S.M., Masoodi F.A., Wani T.A., Ahmad M., Gani A., Ganai S.A. (2015). Physical characteristics, mineral analysis and antioxidant properties of some apricot varieties grown in North India. Cogent Food Agric..

[33] Korekar G., Stobdan T., Arora R., Yadav A., Singh S.B. (2011). Antioxidant capacity and phenolics content of apricot (*Prunus armeniaca* L.) kernel as a function of genotype. Plant Foods Hum. Nutr..

[34] Sochor J., Skutkova H., Babula P., Zitka O., Cernei N., Rop O., Kizek R. (2011). Mathematical evaluation of the amino acid and polyphenol content and antioxidant activities of fruits from different apricot cultivars. Molecules.

[35] Wani S.M., Hussain P.R., Masoodi F.A., Ahmad M., Wani T.A., Gani A., Rather S.A., Suradkar P. (2017). Evaluation of the composition of bioactive compounds and antioxidant activity in fourteen apricot varieties of North India. J. Agric. Sci..

[36] Kara Y., Birinci C. (2024). Usability of the phenolic profile analysis method developed in RP-HPLC-PDA in natural products. J. Apitherapy Nat..

[37] Kara Y., Aybar M., Kolaylı S. (2025). Physicochemical, phenolic, and antioxidant profiles of *Vitis vinifera* L. and *Vitis labrusca* L. grapes from Artvin, Eastern Black Sea Region. J. Food Sci..

[38] Larrigaudière C., Lentheric I., Puy J., Pintó E. (2004). Biochemical characterisation of core browning and brown heart disorders in pear by multivariate analysis. Postharvest Biol. Technol..

[39] Kaiser H.F. (1960). The application of electronic computers to factor analysis. Educ. Psychol. Meas..

[40] Wani S.M., Gull A., Ahad T., Malik A.R., Ganaie T.A., Masoodi F.A., Gani A. (2021). Effect of gum Arabic, xanthan, and carrageenan coatings containing antimicrobial agent on postharvest quality of strawberry: Assessing physicochemical, enzyme activity, and bioactive properties. Int. J. Biol. Macromol..

[41] Silva C.R., Simoni J.A., Collins C.H., Volpe P.L.O. (1999). Ascorbic acid as a standard for iodometric titrations: An analytical experiment for general chemistry. J. Chem. Educ..

[42] Cao S., Yang Z., Zheng Y. (2013). Sugar metabolism in relation to chilling tolerance of loquat fruit. Food Chem..

[43] Slinkard K., Singleton V.L. (1977). Total phenol analysis: Automation and comparison with manual methods. Am. J. Enol. Vitic..

[44] Fukumoto L.R., Mazza G. (2000). Assessing antioxidant and prooxidant activities of phenolic compounds. J. Agric. Food Chem..

[45] Benzie I.F.F., Strain J.J. (1996). The ferric reducing ability of plasma (FRAP) as a measure of “antioxidant power”. Anal. Biochem..

[46] Molyneux P. (2004). The use of the stable free radical diphenylpicrylhydrazyl (DPPH) for estimating antioxidant activity. Songklanakarin J. Sci. Technol..

