# Supplementary material for: Evaluation of Physicochemical Properties, Bioactive Composition, and Antioxidant Activity of Prunus armeniaca L. Cultivars for Functional Food and Nutraceutical Development
Source: Molecules. 2026 Mar 16;31(6):988. doi: 10.3390/molecules31060988 (PMC13028809; doi:10.3390/molecules31060988)
Supplement: Supplementary file 1 [file molecules-31-00988-s001.zip › molecules-4139717-supplementary.pdf]

*molecules*

*Supplementary Materials*

# Evaluation of Physicochemical Properties, Bioactive Composition, and Antioxidant Activity of *Prunus armeniaca* L. Cultivars for Functional Food and Nutraceutical Development

**Ceren Birinci [1], Anna Kurek-Górecka [2,*], Elsevar Asadov [3], Zenon P. Czuba [2] and Sevgi Kolaylı [1,3,*]**

1 Department of Chemistry, Faculty of Science, Karadeniz Technical University, 61080 Trabzon, Türkiye; cerendidar.birinci@gmail.com
2 Department of Microbiology and Immunology, Faculty of Medical Sciences in Zabrze, Medical University of Silesia in Katowice, Jordana 19, 41-808 Zabrze, Poland; zczuba@sum.edu.pl
3 Nakhchivan State University, Nakhchivan AZ7012, Azerbaijan; asadoves1974@gmail.com
* Correspondence: akurekgorecka@sum.edu.pl (A.K.-G.); skolayli@ktu.edu.tr (S.K.)

**Table S1**. Validation parameters (linearity, LOD, LOQ, precision, and recovery) for the HPLC–PDA determination of phenolic compounds. Kara et al. [36]

| Standards | R2 | Limit of Detection (LOD) (µg/mL) | Limit of Quantification (LOQ) (µg/mL) | Relative Error | Recovery (%) |
|---|---|---|---|---|---|
| Gallic acid | 0.999 | 0.043 | 0.142 | 0.007 | 99.296 |
| Protocatechuic acid | 0.999 | 0.062 | 0.205 | 0.008 | 100.838 |
| Chlorogenic acid | 0.999 | 0.034 | 0.115 | 0.018 | 101.757 |
| Catechin hydrate | 0.999 | 0.019 | 0.063 | 0.011 | 98.917 |
| *P*-OH benzoic acid | 0.999 | 0.044 | 0.148 | 0.028 | 102.816 |
| Epicatechin | 0.997 | 0.033 | 0.109 | 0.028 | 102.843 |
| Caffeic acid | 0.999 | 0.072 | 0.239 | 0.027 | 102.716 |
| Vanillic acid | 0.998 | 0.043 | 0.143 | 0.022 | 97.803 |
| Syringic acid | 0.999 | 0.021 | 0.071 | 0.013 | 98.725 |
| *P*-coumaric acid | 0.999 | 0.064 | 0.214 | 0.028 | 102.830 |
| Rutin | 0.999 | 0.047 | 0.156 | 0.004 | 100.417 |
| Ellagic acid | 0.999 | 0.062 | 0.208 | 0.004 | 99.554 |
| Ferulic acid | 0.995 | 0.026 | 0.088 | 0.031 | 103.073 |
| Myricetin | 0.998 | 0.032 | 0.108 | 0.013 | 101.348 |
| Daidzein | 0.999 | 0.050 | 0.166 | 0.007 | 100.719 |
| Luteolin | 0.994 | 0.032 | 0.106 | 0.034 | 103.351 |
| Quercetin | 0.999 | 0.029 | 0.098 | 0.009 | 99.101 |
| *t*-Cinnamic acid | 0.999 | 0.021 | 0.071 | 0.008 | 99.242 |
| Naringenin | 0.996 | 0.026 | 0.087 | 0.008 | 99.181 |
| Apigenin | 0.999 | 0.037 | 0.123 | 0.040 | 103.948 |
| Hesperetin | 0.999 | 0.023 | 0.075 | 0.013 | 101.289 |
| Rhamnetin | 0.996 | 0.028 | 0.094 | 0.014 | 101.445 |

 https://doi.org/10.3390/molecules31060988

| | | | | | |
|---|---|---|---|---|---|
| Chrysin | 0.999 | 0.027 | 0.091 | 0.009 | 99.146 |
| Pinocembrin | 0.999 | 0.037 | 0.123 | 0.007 | 99.303 |
| CAPE | 0.999 | 0.025 | 0.083 | 0.035 | 103.456 |
| Galangin | 0.999 | 0.023 | 0.077 | 0.036 | 103.647 |

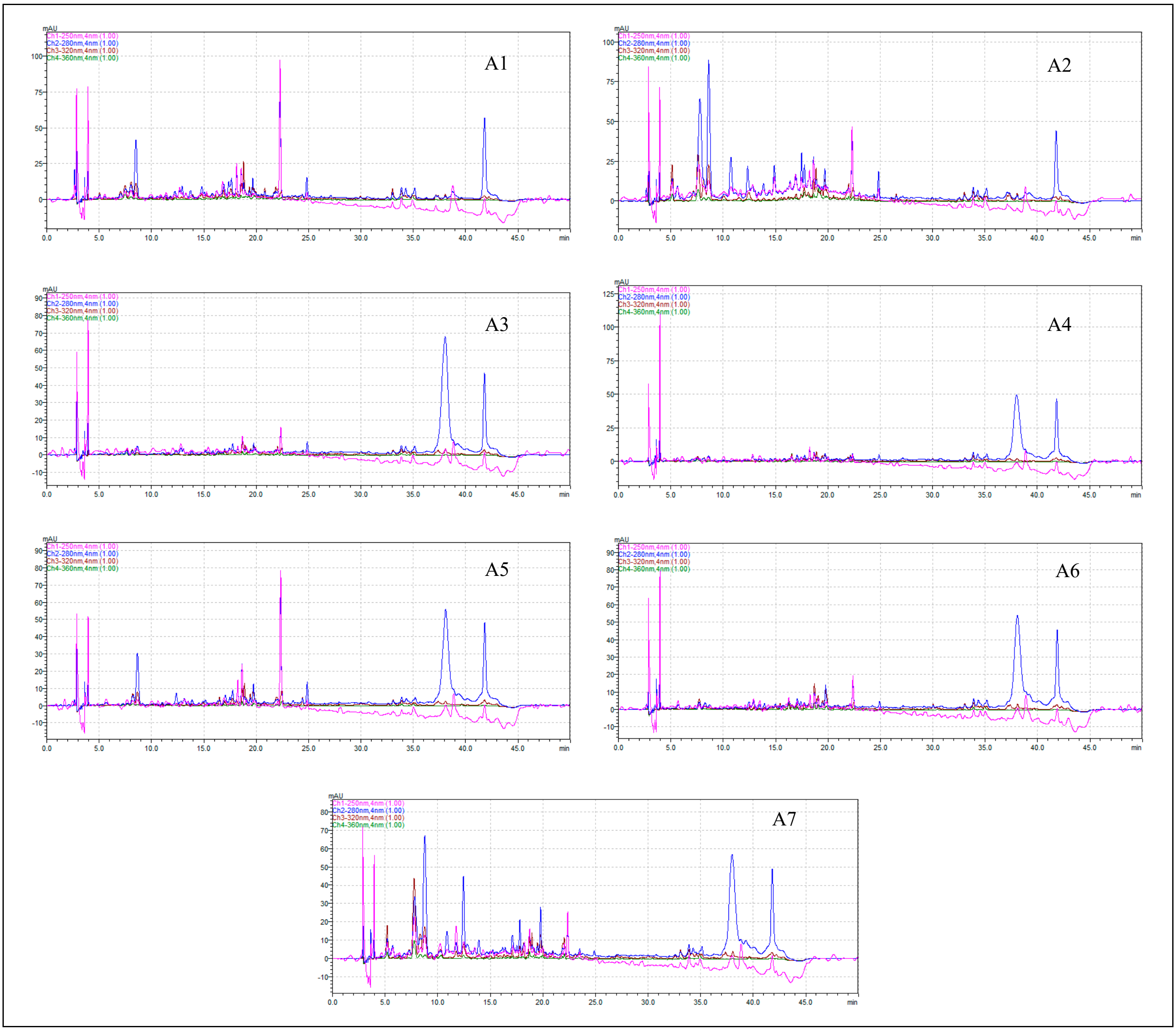


**Figure S1**. HPLC chromatograms of phenolic compounds identified in apricot samples

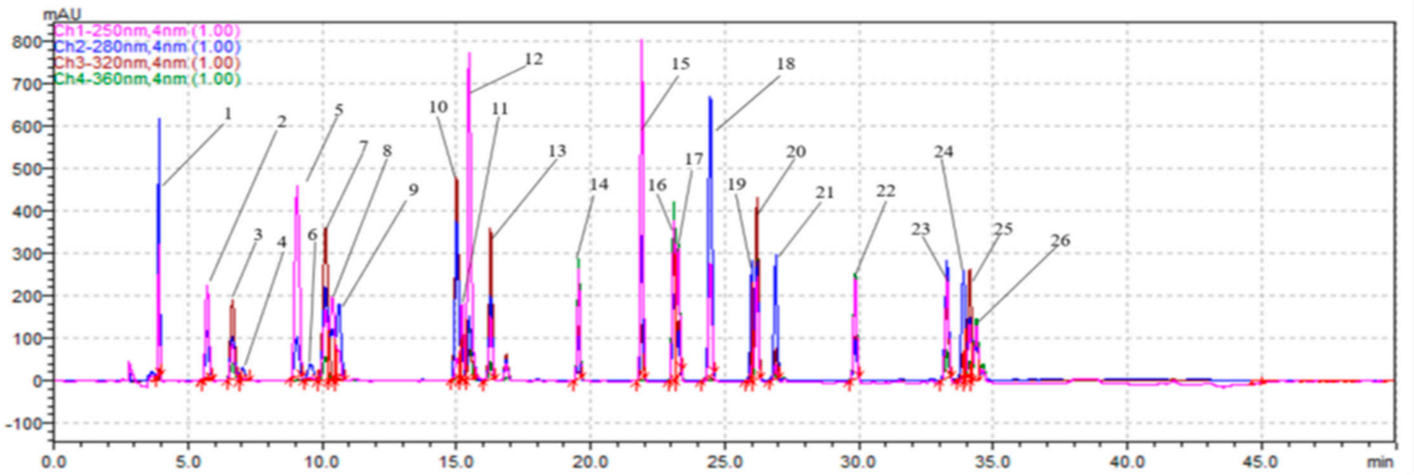


**Figure S2.** Phenolic Standard Chromatogram.

1. Gallic acid, 2. Protocatechuic acid, 3. Chlorogenic acid, 4. Catechin hydrate, 5. *p*-OH Benzoic acid, 6. Epicatechin, 7. Caffeic acid, 8. Vanillic acid, 9. Syringic acid, 10. *p*-Coumaric acid, 11. Rutin, 12. Ellagic acid, 13. Ferulic acid, 14. Myricetin, 15. Daidzein, 16. Luteolin, 17. Quercetin, 18. *t*-Cinnamic acid, 19. Naringenin, 20. Apigenin, 21. Hesperetin, 22. Rhamnetin, 23. Chrysin, 24. Pinocembrin, 25. CAPE, 26. Galangin Kara et al. [36]

| **Code** | **Image** | **Collected area**<br>**Local name** |
|---|---|---|
| **A-1** | 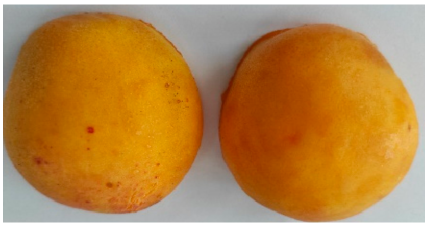 | Nakhchivan<br>Şeftali şalax |
| **A-2** | 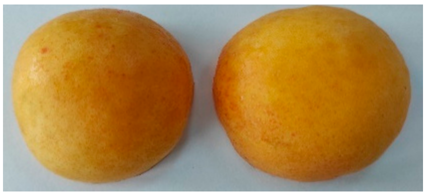 | Nakhchivan<br>Adi şalax |
| **A-3** | 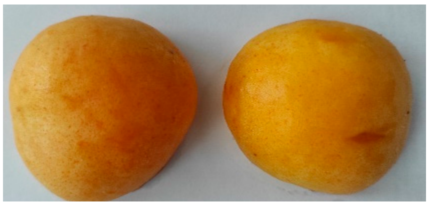 | Nakhchivan<br>Badami |
| **A-4** | 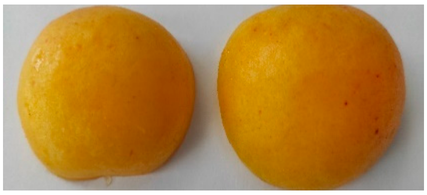 | Nakhchivan<br>Balyarim |
| **A-5** | 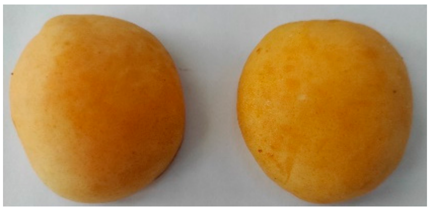 | Türkiye<br>Iğdır |
| **A-6** | 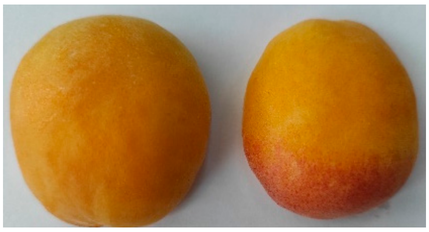 | Türkiye<br>Malatya<br>(Hacıoğlu) |

https://doi.org/10.3390/molecules31060988

| | | |
|---|---|---|
| **A-7** | 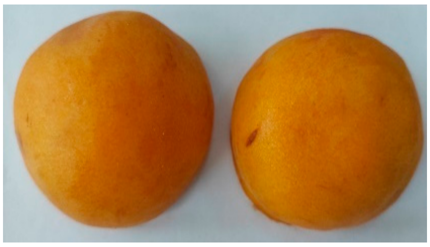 | Türkiye<br>Amasya |

**Figure S3.** The image and names of the apricot's fruits

https://doi.org/10.3390/molecules31060988
